# Generation of Backward-Looking Complex Reflections for a Motivational Interviewing–Based Smoking Cessation Chatbot Using GPT-4: Algorithm Development and Validation

**DOI:** 10.2196/53778

**Published:** 2024-09-26

**Authors:** Ash Tanuj Kumar, Cindy Wang, Alec Dong, Jonathan Rose

**Affiliations:** 1 Faculty of Applied Science & Engineering, University of Toronto, Toronto, ON, Canada; 2 The Edward S Rogers Sr Department of Electrical and Computer Engineering, University of Toronto, 10 King's College RoadToronto, ON, M5S 3G4, Canada, +1 416-978-6992

**Keywords:** motivational interviewing, smoking cessation, therapy, automated therapy, natural language processing, large language models, GPT-4, chatbot, dialogue agent, reflections, reflection generation, smoking, cessation, ChatGPT, smokers, smoker, effectiveness, messages

## Abstract

**Background:**

Motivational interviewing (MI) is a therapeutic technique that has been successful in helping smokers reduce smoking but has limited accessibility due to the high cost and low availability of clinicians. To address this, the MIBot project has sought to develop a chatbot that emulates an MI session with a client with the specific goal of moving an ambivalent smoker toward the direction of quitting. One key element of an MI conversation is reflective listening, where a therapist expresses their understanding of what the client has said by uttering a *reflection* that encourages the client to continue their thought process. *Complex* reflections link the client’s responses to relevant ideas and facts to enhance this contemplation. Backward-looking complex reflections (BLCRs) link the client’s most recent response to a relevant selection of the client’s previous statements. Our current chatbot can generate complex reflections**—**but not BLCRs—using large language models (LLMs) such as GPT-2, which allows the generation of unique, human-like messages customized to client responses. Recent advancements in these models, such as the introduction of GPT-4, provide a novel way to generate complex text by feeding the models instructions and conversational history directly, making this a promising approach to generate BLCRs.

**Objective:**

This study aims to develop a method to generate BLCRs for an MI-based smoking cessation chatbot and to measure the method’s effectiveness.

**Methods:**

LLMs such as GPT-4 can be stimulated to produce specific types of responses to their inputs by “asking” them with an English-based description of the desired output. These descriptions are called *prompts*, and the goal of writing a description that causes an LLM to generate the required output is termed *prompt engineering*. We evolved an instruction to prompt GPT-4 to generate a BLCR, given the portions of the transcript of the conversation up to the point where the reflection was needed. The approach was tested on 50 previously collected MIBot transcripts of conversations with smokers and was used to generate a total of 150 reflections. The quality of the reflections was rated on a 4-point scale by 3 independent raters to determine whether they met specific criteria for acceptability.

**Results:**

Of the 150 generated reflections, 132 (88%) met the level of acceptability. The remaining 18 (12%) had one or more flaws that made them inappropriate as BLCRs. The 3 raters had pairwise agreement on 80% to 88% of these scores.

**Conclusions:**

The method presented to generate BLCRs is good enough to be used as one source of reflections in an MI-style conversation but would need an automatic checker to eliminate the unacceptable ones. This work illustrates the power of the new LLMs to generate therapeutic client-specific responses under the command of a language-based specification.

## Introduction

### Background

Smoking cessation therapists have long used the motivational interviewing (MI) talk therapy to guide clients toward positive behavioral change [1]. MI engages clients in a structured conversation that encourages them to contemplate their behavior more deeply and motivates them to change it. MI has been shown to be successful in helping clients reduce or quit their smoking habits [2], but the availability of MI-trained clinicians is limited to hospitals and medical centers, and MI therapy is usually only initiated after a smoking-related health issue occurs [3]. These restrictions make it difficult for smokers to access therapy outside of medical centers and occur too late to have a preventative effect.

Our research seeks to automate the therapist side of an MI conversation which, if successful, could broaden access to care at a population level. We have been developing a chatbot, called MIBot [4], whose purpose is to move ambivalent smokers toward the direction of quitting. MIBot is being developed by an interdisciplinary research collaboration among expert MI-trained clinicians, social scientists, and computer engineers. The initial version of the MIBot chatbot guides the client through a fairly simple MI conversation by combining scripted interactions with context-specific responses generated by natural language models, based on elements of the MI approach.

The focus of the initial version of the MIBot chatbot is on one core skill of MI: reflective listening [1], in which the chatbot provides reflections on what the client has most recently said. In general, reflections are meant to express the therapist’s current understanding of the client’s most recent response and invite the client to continue further contemplation of their behavior. Reflections can be simple or complex [1]. A simple reflection rephrases a client’s response, sending the message that the response was understood and inviting the client to continue. A complex reflection attempts to infer relevant information about the client from the client’s utterance by linking the client’s response to relevant facts or ideas. A good quality complex reflection may further infer something about the emotional state of the client through their utterance.

In a complex reflection, when these relevant facts come from a client’s earlier responses in the conversation, we call this a backward-looking complex reflection (BLCR). Preferably, a BLCR does not simply summarize all the past conversational information in order but is composed of the information that is sensible for the context. Textbox 1 shows an example of a conversation in which the final statement by the therapist is a BLCR.

Textbox 1. **Example motivational interviewing conversation in which the last utterance by the therapist is a backward-looking complex reflection.**

Therapist: What is one thing you like about smoking?

Client: It makes me have less stress and keeps me connected to my friends.

Therapist: What is one thing you dislike about smoking?

Client: It leaves bad breath.

Therapist: What is one thing about your smoking addiction that you would like to change?

Client: I would like to reduce smoking.

Therapist: [backward-looking complex reflection] It seems like you want to reduce your smoking, which might help your concern about bad breath

The initial MIBot chatbot [4] only generates reflections using the client’s most recent utterance and does not make use of prior utterances. The ability to generate BLCRs can expand the chatbot’s options for generating context-appropriate complex reflections.

The goal of this work is to develop and evaluate a method to automatically generate BLCRs given a prior conversation. It has become possible to do this kind of generation through recent dramatically powerful advancements in natural language processing [5], and more specifically the most recent large language models (LLMs) from GPT-3.5 and later [6-8].

LLMs are language models which take text as input and generate textual output. GPT-4, an LLM introduced in March 2023, has significantly improved capability to generate text to satisfy particular requirements compared to previous LLMs [6-9]. One way to use GPT-4 is to write a *prompt*, which is a language-based instruction that literally tells the model the processing that is desired [9]. This processing is potentially anything that can be described in language, which is a truly remarkable, new capability that will have many applications. We describe a method for developing the prompts needed to “tell” the model to create BLCRs.

This paper is organized as follows: the *Prior Work* section introduces MI, GPT-4, and the relevant parts of the MIBot project that we build on. The *Methods* section describes the prompt developed to generate a BLCR, the specific structure of the input to GPT-4, the rating scale developed to assess when a BLCR is acceptable, the experimental procedure to test the acceptability of BLCRs generated by the prompt, and the data used to test this procedure. The *Results* section provides the evaluation, and the *Discussion* section interprets the results of the experiment and lists limitations. The *Conclusions* section suggests avenues for further work.

### Prior Work

#### Motivational Interviewing

MI is a therapeutic technique in which a therapist engages in a conversation to guide and motivate clients who are ambivalent about their behaviors to move toward changing them [1]. These guided conversations use 4 MI core skills: asking open-ended questions, providing reflections, affirmations, and summarization. In an MI conversation, the therapist will typically begin with an open-ended question, listen to the client’s response, and reply with 1 of the other 3 core skill types, depending on the circumstances and the direction the therapist wishes to guide the conversation.

While all 4 core skill types are integral to a successful MI, we focus on the role of reflections and the related reflective listening. Reflective listening requires the therapist to listen to what the client has most recently said and formulate a response*—*called a reflection*—*that displays the therapist’s understanding while also guiding the conversation. The content of a reflection depends on the current context of the conversation. Reflections can be divided into 2 types: simple reflections and complex reflections. Simple reflections restate the client’s response, typically using different words, so that the therapist and client can establish that they are on the same page. Complex reflections allow the therapist to link what the client has most recently said to other facts or information about the client’s life and emotional state, usually providing some kind of inference. Complex reflections are used to guide the conversation toward new topics.

MI has been shown to be a successful therapy for moving clients toward reducing their smoking habits [2], and reflections in particular have been correlated with high perceived support for patient autonomy in MI sessions [10].

#### LLMs and GPT-4

LLMs are digital models of natural language that are able to generate text from an input by autoregressively predicting the next word in a given sequence [6]. These models learn how to predict semantically and syntactically reasonable words by being trained to “fill in the blanks” on large amounts of diverse human-written text, which encompass questions and answers, web-based conversations, informative articles, and other kinds of digitized text. The wide range of data that LLMs are trained on have made them effective systems for generating solutions to various problems in the domain of natural language processing, such as answering questions, summarizing long text, and conversational dialogue generation [7].

The GPT (Generative Pretrained Transformer) family of LLMs has proven to be state of the art in a number of general-purpose tasks [8]. ChatGPT and the related GPT-4 [9] model can generate human-like text and answer questions correctly to the point that it has successfully passed m any professional and academic examinations [9].

Due to the size of the model and the large amounts of human-produced textual data it is trained on, 1 emergent characteristic of the GPT-4 is its ability to answer questions and interpret human-readable text to follow instructions. This has led researchers to try and directly “ask” GPT-4 to generate some kind of desired text given some input. The study of ways to ask GPT-4 to generate desired text is a newly emerging field called prompt engineering. Thus, this asking process is called *prompting* a GPT-4 model, and these “asks” are typically called *prompts* [9,11].

A prompt usually consists of a *request* of the model to generate or process some desired text, usually followed by requirements that the generated text must satisfy or instructions that tell the model how to generate this text [11]. The request can optionally be followed by an *input*, with the goal that the model will use the input to process and generate the requested text (Table 1).

**Table 1.** Prompt example and generated result. The request is the first sentence, and the input is the italicized second line. The request and input message can be tested live on the OpenAI playground by copy and pasting the entire prompt text [9].

	Prompt example and GPT-4–generated result
Prompt (request and input)	Given the Keywords below, write a paragraph that incorporates them into a story about a princess on the moon.Keywords: spaceship, cat, star, Pluto, Brazil
GPT-4	*Once upon a time, there lived a princess, unlike any other, on the moon. She had a spaceship that allowed her to travel across galaxies, making frequent visits to far-off planets. It was on one such trip that she found a stray cat wandering amidst the frozen nitrogen landscapes of Pluto, looking as lost and lonely as a star flickering bleakly in an infinite sea of black. She couldn’t bear its sight and decided to take this feline friend along on her adventures. To cheer the cat up, they took spontaneous trips to Earth, her favorite being Brazil. Their spaceship would hover quietly over the vibrant Amazon forest as the princess and her cat would gaze down at the colorful montage of life. The brilliant night sky with millions of stars reflected in the cat’s eyes reminded the princess that, just like the starlight, even the loneliest souls could brighten up the darkest corners of the universe.*

GPT-3– and GPT-4–based prompting has been shown to be highly effective in generating text to solve various natural language processing tasks [9,11,12] and has already found applications in a diverse set of technical fields. However, a prompted GPT model does not always produce factually correct answers [9,11,12]. In addition, a prompted GPT model is not deterministic, and a single prompt may produce different texts each time that a prompt is used to generate a completion [11,13]. Recent research on prompt engineering has produced new methods to structure prompts for generating satisfactory texts [13].

The ability to prompt is not restricted by the architecture of GPT-3 or GPT-4. Prompting is possible with any LLM of similar structure, and the difference in output depends on how much knowledge and prediction capability has been retained by an LLM. Thus, while our work specifically used GPT-4, this paper’s method can be used with any LLM, including future improvements on GPT-4, and we will indicate this by referring to LLMs broadly in our methods and discussions.

#### Existing MI Smoking Cessation Chatbots and the MIBot Project

The research and development of MI-based chatbots across several therapeutic domains remains an open problem, with numerous approaches incorporating different natural language processing techniques, and nothing yet deployed in a commercial or therapeutic context for mass adoption. For MI focusing on smoking cessation, several research teams have independently developed chatbots that have been tested and evaluated on experimental study participants. Our particular work has focused on an early step in smoking cessation, which is moving ambivalent smokers toward the decision to quit smoking.

Almusharraf et al [14] designed an MI chatbot, which used predefined answers in a scripted conversation and measured its effectiveness on clients’ confidence to quit smoking with an 11-point scale. After testing this method on 97 participants, they found that the average confidence among clients to quit smoking increased by 0.8 (*P*<.001 via paired 1-tailed *t* test) 1 week after the conversation. The scripted nature of these MI conversations, with answers not unique to clients’ responses, was suggested as a future point of improvement to investigate further.

Independently, He et al [15] sought to investigate whether chatbots using MI techniques had any differing effects from neutral chatbots. They designed 2 chatbots*—*an MI-based chatbot and a neutral, affirming chatbot*—*and found that while there were no significant differences in clients’ reception of the 2 chatbots, both chatbots increased the clients’ motivations to quit smoking. The conclusions of He et al [15] combined with the results of Almusharraf et al [14] indicate that nonscripted responses from chatbots may be better received.

The text produced by generative models are an alternative to scripted responses, and Shen et al [16] displayed how generative models could generate reflections dependent on context. Using a GPT-2–based architecture, they created unique, context-dependent generative responses by incorporating a combination of client and therapist utterances from an existing dialogue history, and drawing from a database of previous transcripts to help select between context-relevant responses based on semantic similarity. These generated reflections were compared to a seq2seq model baseline, an older model of conditional text generation that is not LLM based, and human evaluation using a 5-point Likert scale for absolute effectiveness. The generated reflections produced by this system were considered improvements over the baseline model using standard metrics such as the Recall-Oriented Understudy for Gisting Evaluation (ROUGE) score and, in terms of absolute effectiveness, were on-par or above ground truth reference reflections. These results indicate that custom reflections from generative models may be effective for MI-based smoking cessation chatbots to increase users’ confidence and motivation in quitting smoking.

To explore this possibility, Brown et al [4] have been iteratively developing MIBot, an MI-based smoking cessation chatbot that uses GPT-2 to generate custom reflections. They tested 3 versions of the chatbot*—*labeled v5.0, v5.1, and v5.2*—*on independent groups of recruited smokers to measure the effect of GPT-2–based generative reflections on moving smokers towards changing their smoking habits. They also used a version of the chatbot that did not generate reflections*—*v4.7*—*for comparison. MIBot v5.0, v5.1, and v5.2 asked 5 core questions, shown in Textbox 2 in sequence, expected a participant response after each question, and used a pretrained GPT-2 model to generate a custom reflection. MIBot v5.2 added extra secondary questions after questions 1 and 2 to allow participants to follow-up with their initial responses to a core question, and a specific version of question 4 if the answer to question 3 was to reduce smoking. MIBot v4.7 also asked these questions, but responded with “thank you” to each response rather than generating a reflection.

Textbox 2. **The 5 motivational interviewing conversational questions in the MIBot v5.2 conversation used in this paper.**

1. What is one thing you like about smoking?
2. What is one thing you dislike about smoking?
3. What is one thing about your smoking addiction that you would like to change?
4. What will your life look like once you make this change?
5. What is one step you need to make this change?

The effect of MIBot versions on readiness to quit was measured using a numerical scale called the Readiness Ruler [17]. Here, each participant was asked to rate their confidence, importance, and readiness to quit smoking from 0 to 10, with 10 indicating the highest value. Participants were asked to fill out the Readiness Ruler 3 times: just before, immediately after, and 1 week after the conversation with MIBot. Participants were also asked to score the perceived empathy of MIBot through the CARES (Consultation and Relational Empathy Survey) metric, a validated tool used to measure the perceived empathy of a health care interaction by asking a participant 10 statements that are each rated using a 6-point Likert scale [18].

Brown et al [4] found that there were statistically significant increases in participant confidence to quit smoking across all four chatbots 1 week after the conversation, with no statistically significant differences between them. This finding agreed with He et al’s [15] results, and Brown et al [4] posited that asking questions may be enough to evoke an impact on confidence to quit. Version v5.2 did display statistically significant increases in importance and readiness to quit smoking when the other versions did not. In addition, v5.2 did exhibit a statistically significant increase in perceived empathy compared to v4.7 (*P*=.004) on the CARE scale. Both results were in contrast to He et al’s [15] findings that there were no statistically significant differences between neutral and MI-style chatbot conversations, and Brown et al [4] postulated that this may be due to the effect of v5.2’s LLM-based generative reflections.

MIBot v5.0, v5.1, and v5.2 generate GPT-2–based reflections that only use a participant’s latest response. This precludes the generation of complex reflections that can refer to earlier responses in a conversation, which are the essential element of the BLCRs that are the focus of this paper. This work builds upon Brown et al’s [4] work by creating and evaluating a method to generate BLCRs using GPT-4.

## Methods

### Overview

In this section, we describe the structure of the method used to generate BLCRs, the set of data we test our BLCR generation method on and how the resulting BLCRs are assessed.

### Ethical Considerations

Ethical standards and approval directly follow those of Brown et al [4] as per the use of the data in the experiments described in that paper. The research used to acquire that data was approved by the University of Toronto Research Ethics Board under protocol number 35567, amended June 29, 2022, and all participants provided consent before participating in the Brown et al [4] study.

### BLCR Generation Structure

In a chatbot conversation with a client, the client’s latest and previous responses, along with the questions that were asked to evoke those responses, are packaged into a text called the *client message input*. A set of instructions, called the *BLCR prompt*, tells an LLM how to generate a BLCR from the client message input. These 2 texts are used together to generate a BLCR.

### Client Message Input

The client message input (Textbox 3) consists of (1) conversation—the sequence of therapist questions and client responses up to the client’s response right before the therapist’s latest question—and (2) latest question-response—the therapist’s latest question and the client’s latest response.

Textbox 3. **A sample client message input.**

**Conversation:**

Therapist: What is one thing you like about smoking?

Client: It makes me to be more relaxed and releases my tension levels

Therapist: What is one thing you dislike about smoking?

Client: It would be the number of cigarettes I smoke a day plus the affordability of cigarettes theses days

Therapist: What is one thing about your smoking habit that you would like to change?

Client: The number or quantity I smoke a week

Therapist: What will your life look like when you make this change?

Client: If I can reduce by smoking 2 cigarettes a day and I would have some extra cash to do other things

**Latest Question-Response:**

Therapist: What are the steps you need to make this change?

Client: I need to probably set a smoking schedule that I need to stick too and also find a hobby to keep me distracted from my cravings

**Backward-looking complex reflection:**

The client message input is unique to each client response, and so changes on every client response. An LLM processes this input to generate a BLCR by first processing the instructions given in the BLCR prompt.

### Prompt Design

The BLCR prompt, shown in Textbox 4, consists of (1) a request to generate a BLCR meeting the standards of MI, using terms presented in the client message input (see *Client Message Input* section); (2) a description of a complex reflection, taken from Miller and Rollnick [1]; (3) constraints and criteria to ensure the generated text meets the criteria of a complex reflection; (4) constraints and criteria to ensure the generated text meets the criteria of a BLCR; and (5) repetition of the request to generate a BLCR, given the above constraints and criteria.

The BLCR prompt is the same regardless of the client input message used. The BLCR prompt draws upon an LLM’s implicit domain knowledge of MI [4,11], combined with a specific definition of a complex reflection, and constraints and criteria on what the output must follow to be an acceptable BLCR. For each client message input, an LLM can use the BLCR prompt’s guidelines to generate a BLCR.

Textbox 4. **The full backward-looking complex reflection prompt.**

Generate a "backward-looking complex reflection" on the "Latest Question-Response" that meets the standards for Motivational Interviewing from the given "Conversation" about smoking cessation.

Refer to the following operational definition of a complex reflection in the context of Motivational Interviewing (MI):

Reflective listening statements are made by the clinician in response to client statements. A reflection may introduce new meaning or material, but it essentially captures and returns to clients something about what they have just said. Reflections are further categorized as simple or complex reflections.

Complex reflections typically add substantial meaning or emphasis to what the client has said. These reflections serve the purpose of conveying a deeper or more complex picture of what the client has said. Sometimes the clinician may choose to emphasize a particular part of what the client has said to make a point or take the conversation in a different direction. Clinicians may add subtle or very obvious content to the client's words, or they may combine statements from the client to form complex summaries.

A complex reflection has these hard constraints:

1. A complex reflection must be a statement and not a question.
2. A complex reflection must not give advice or information without permission, even if this advice is helpful.
3. A complex reflection must not direct the client by giving orders or commands.
4. A complex reflection must not disagree or challenge what the client has said.
5. A complex reflection must not incentivize people to smoke more, or discourage people from quitting smoking.
6. A complex reflection must not be factually wrong about smoking.
7. A complex reflection must be grammatically correct.

Here are some additional hard constraints for backward-looking complex reflections:

1. A backward-looking complex reflection must directly reference the Client statement and the Therapist question it is responding to in the Latest Question-Response.
2. A backward-looking complex reflection must include only one piece of extra information from earlier client statements in the Conversation.
3. A backward-looking complex reflection must not summarize the conversation.
4. A backward-looking complex reflection must use what the client has said in the last client statement, and the information from earlier client statements, and infer something about the client.

Given all the context above, generate a backward-looking complex reflection on the "Latest Question-Response" from the given "Conversation" that meets the Motivational Interviewing criteria of a complex reflection and satisfies all above hard constraints.

The BLCR prompt was created through an iterative process. Starting with an initial description was set of rules describing a BLCR and the requirements to generate a BLCR. This initial prompt was used to generate reflections on preexisting conversational data from prior conversations. These reflections were evaluated using the scale described in the *Evaluation of Quality of a BLCR* section. The prompt was subsequently revised to improve the responses, and the method attempted again on another set of independent conversational data. The revisions consisted of additional constraints and guidance, written in English, to address the shortcomings of the generated reflections. This iterative process continued until a prompt of sufficiently high evaluation score of the generated reflections was was achieved. The following sections describe both the data and the scale used.

### Data

To test the BLCR prompt and client message inputs on real conversational data, 50 conversations were randomly selected from the MIBot version5.1 experiment data [4]. Each conversation consisted of the 5 MIBot core questions shown in (Textbox 2), along with their respective participant responses. As described in Brown et al [4], the participants were 50 anonymous volunteers from the Prolific platform who self-selected based on being current smokers. All 50 participants wrote their responses in text via the MIBot text-based chat interface. Multimedia Appendix 1 provides a sample conversation. Using the BLCR prompt and client message input, BLCRs would be generated for responses to Q3, Q4, and Q5 for each conversation, giving a total of 150 candidate BLCRs to assess.

### Evaluation of Quality of a BLCR

A rating scale was developed to numerically evaluate the quality of a BLCR. This scale allows one to determine whether a BLCR is *acceptable*, that is, it meets the definition of a BLCR described in the *Prior Work* section.

The BLCR rating scale (Textbox 5) is an ordinal scale where higher number ratings successively include and build upon lower number ratings. If a BLCR achieves a rating of 3, this means it meets the criteria of 1 (referencing a client’s latest response), 2 (referencing previous information in the conversation), and 3 (makes an inference about the client using present and past information). Satisfying these 3 requirements meets the definition of a BLCR as defined in the *Prior Work* section; therefore, we call any BLCRs rated 3 or greater acceptable BLCRs. A further rating of 4 is included to meet the preference for a “good” BLCR, which does not summarize the previous contents of the conversation, an optional condition that was deemed useful for indicating an unambiguous BLCR that exceeds the minimum acceptability requirements.

Textbox 5. **The backward-looking complex reflection rating scale.**

**1: does the output reference the client’s latest response somewhere?**

- the output contains 1 or more references to the client’s latest response

**2: 1 + does the output reference some extra information from earlier in the conversation?**

- the output contains 1 or more references to 1 or more previous client responses

**3: 2 + does the output make an inference about the client using information in criteria 1 and 2?**

- the output generates 1 or more novel assumptions about the client using information in 1 and 2

**4: 3 + is the output not summarizing the sequence of the conversation word for word?**

- the output does not repeat the information in each client response in sequence

**Criteria to accept as a backward-looking complex reflection (score a 1 [True]):** it is rated 3 or greater on the above rating scale.

A Python script was written to parse 50 conversations and build a formatted client message input for every Q3, Q4, and Q5 conversational sequence, creating 150 total inputs. These were fed to an LLM alongside the BLCR prompt, and the LLM generated 150 candidate BLCRs.

Three human raters were deployed to use the criteria of the BLCR Rating Scale to independently score all 150 generated BLCRs as acceptable or unacceptable. Using a binary score, an acceptable BLCR was scored 1 (true) if it received a rating of 3 or greater on the BLCR Rating Scale, while an unacceptable BLCR was scored 0 (false). The binary scoring was used to determine the *acceptability*: the percentage of accepted BLCRs among all generated BLCRs. The interrater reliability between the binary scores of the 3 raters was assessed using percent agreement and the calculation of Cohen *κ*. This metric was chosen specifically to measure interrater reliability with an ordinal scale, and was chosen instead of a similar metric such as Fleiss *κ* due to the latter’s unsuitability in a case where all raters rate all items, which is the case for this BLCR assessment experiment [19].

## Results

### Overview

This section reports the fraction of the BLCRs generated using the evaluation method described in the *Methods* section that were deemed acceptable by each of the 3 human raters. The first section reports the percentage of accepted BLCRs between the 3 raters and between the 3 questions, along with a breakdown of the frequency of ranking scores per question and rater. The second section reports the interrater reliability between 3 pairs of the 3 raters (rater 1 and rater 2, rater 1 and rater 3, and rater 2 and rater 3) using percent agreement, with a brief discussion on the *κ* results.

### BLCR Acceptability Statistics

Table 2 displays the percentage of BLCRs meeting the BLCR rating criteria as acceptable (BLCR rating of 3 or greater) broken down by the rater and the question. Table 3 displays the frequency of rating ranks broken down by question and by rater.

**Table 2.** Percentage of backward-looking complex reflections deemed acceptable by question and rater.

	Q3 (n=50)	Q4 (n=50)	Q5 (n=50)	Total (N=150)
Rater 1 (%)	92	90	96	93
Rater 2 (%)	73	90	88	84
Rater 3 (%)	90	88	86	88
Average acceptance (%)	85 (10)	89 (1)	90 (5)	88 (5)

**Table 3.** Frequency of rating by question and rater.

Question and rater		Rating, n				
		0	1	2	3	4
**Q3**						
	Rater 1	4	0	0	3	44
	Rater 2	1	0	13	2	35
	Rater 3	2	0	3	6	40
**Q4**						
	Rater 1	1	2	2	0	46
	Rater 2	1	0	4	0	46
	Rater 3	1	0	5	0	45
**Q5**						
	Rater 1	1	0	1	0	49
	Rater 2	1	0	5	2	43
	Rater 3	3	2	2	0	43
**Total (all questions combined)**						
	Rater 1	6	2	3	3	139
	Rater 2	3	0	22	4	124
	Rater 3	6	2	10	6	129

Table 2 breaks down the percent of acceptable BLCRs by rater and question, and the total column indicates the percent of BLCRs scored acceptable across all 150 responses by a single rater. The percentages in parentheses indicate the SD of the acceptability percentage.

The combination of high acceptability (Table 2) and high frequency of “4” ratings (Table 3) indicates that the majority of BLCRs generated by this method were considered “good” among all 3 raters. This is an indication that the LLM GPT-4 is highly capable of generating a BLCR. Multimedia Appendices 2 and 3 graph the frequencies of rating by question and rater, with both indicating a large skew toward “4” ratings.

### Interrater Reliability

To assess the agreement of the results provided in Tables1  2, Table 4 displays the percent agreement and Cohen *κ* for each rater pair. All 3 raters agreed on results at least 80% of the time.

**Table 4.** Percent agreement and Cohen *κ* for rater pairs.

	Rater 1, rater 2	Rater 1, rater 3	Rater 2, rater 3
Agreement (%)	84	88	80
Cohen *κ*	0.26	0.36	0.16

## Discussion

### Principal Findings

Altogether, the combination of high “4” frequency and a rating agreement of 80% and above indicates that this BLCR generation method can be expected to produce “good” BLCRs in the large majority of cases. In comparison, the *κ* values (Table 3) indicated weak to fair agreement between all 3 pairs of raters, based on standard interpretation criteria of *κ*. The discrepancy between high percentage agreement and weak to fair *κ* may be due to the majority of BLCRs being rated “4” by all 3 raters. The lack of contrastive negative examples (very few generated BLCRs that were rated 0, 1, or 2) skews the calculation of *κ* toward treating the labeling of widespread agreement as random chance. Therefore, percent agreement is thought to be a more realistic assessment of effectiveness in this context.

Multimedia Appendix 1 contains an example of a real conversation from Brown et al [4], with Brown et al’s [4] reflections (labeled MIBot [data]) and BLCRs generated by this paper’s method (labeled MIBot [BLCR]) below those reflections. Overall, the BLCRs generated successfully iterate on Brown et al’s [4] provided reflections by better incorporating direct reflections on responses and linkages to previous responses to make inferences. A high-quality MI reflection would further infer about the emotional state of the client, and while the generated BLCRs are able to make rudimentary inferences about the mental state of the client (“it seems that…”), more work may be necessary to turn these inferences into those of emotional states. The high percentage of accepted BLCRs shows promise in prompt-based methods being an effective technique for MIBot to generate complex reflections that incorporate information from the past.

### Limitations

The prompt-based BLCR generation method is restricted to MI conversations for smoking cessation and has only been tested in the context of 5-question MIBot conversations. Beyond this scope, this work may not generalize to other MI smoking cessation therapeutic contexts without changes to the prompt. However, the structure of the prompt itself is not specific to the data or the situation. The prompt can in theory be modified to remove references to smoking cessation and replace these with references to other domains, potentially offering a degree of domain generalizability across different subjects of MI therapy beyond smoking cessation. GPT-4 was the LLM model used in this work, but this method is applicable to any LLM model in theory. Newer LLM models, including future GPT models, may provide more robust results.

### Conclusions

This paper presented a method to use an LLM-based prompt to generate BLCRs for a version of MIBot’s MI smoking cessation conversation. It provided a definition of a BLCR, a prompt used to generate BLCRs, and a BLCR rating scale to assess whether a BLCR is acceptable. We found that 88% (n=150) of the generated BLCRs were deemed acceptable. This paper extends the work of Brown et al [4] by providing a method to generate complex reflections that incorporate information from earlier in the conversation, and uses GPT-4’s strong text-generation capability rather than GPT-2.

Future work may build upon the definitions and methods introduced by this paper in three ways. First, the definition of a BLCR and the BLCR rating scale may be further refined to provide an accurate conceptual model of what the BLCR is trying to capture in a MI conversation. Second, the BLCR’s prompt method can be adjusted to different MI therapeutic contexts beyond smoking cessation or refined to be more accurate for the smoking cessation context. Finally, the BLCR prompt method can be incorporated into MIBot, and its generated BLCRs can be assessed qualitatively and quantitatively in live experimental conversations.

## Supplementary material

10.2196/53778 Multimedia Appendix 1 Example of a MIBot v5.1 conversation. Generated reflections are italicized. Reflections marked "mibot (data)" are from the original dataset. Reflections marked "mibot (blcr)" are generated from this paper's prompting method. Both are provided for comparison.

10.2196/53778 Multimedia Appendix 2 Frequency of backward-looking complex reflection rating score by question.

10.2196/53778 Multimedia Appendix 3 Frequency of backward-looking complex reflection rating score by rater.

## Abbreviations

BLCR: backward-looking complex reflection
CARES: Consultation and Relational Empathy Survey
GPT: Generative Pretrained Transformer
LLM: large language model
MI: motivational interviewing
ROUGE: Recall-Oriented Understudy for Gisting Evaluation
