# Supplementary material for: Generation of Backward-Looking Complex Reflections for a Motivational Interviewing–Based Smoking Cessation Chatbot Using GPT-4: Algorithm Development and Validation
Source: JMIR Ment Health. 2024 Sep 26;11:e53778. doi: 10.2196/53778 (PMC11448290; doi:10.2196/53778)
Supplement: Multimedia Appendix 1 [file mental-v11-e53778-s001.docx]

**Appendix A**

**Example of a MIBot v5.1 conversation.** **Generated reflections are italicized.** Reflections marked **mibot (data)** are from the **original dataset**, while **mibot (blcr)** are generated by the **BLCR prompting method**. Both are provided for comparison.

| **mibot** | Hello! My name is MIBot, I am a computer chatbot and part of a research project at the University of Toronto. I was made by a group of researchers and doctors, with the goal of exploring your smoking behaviours. |
| --- | --- |
| **mibot** | I will ask a few questions, listen to your answers, and then respond. |
| **mibot** | However, I am learning how to have this conversation. So, I may ask you if the things I say make sense. |
| **mibot** | Would you like to chat with me about your smoking? |
| **client** | Sure |
| **mibot** | Great! |
| **mibot** | To start, what is the thing you like most about smoking? |
| **client** | stress relief |
| **mibot (data)** | *You enjoy the relief that smoking provides you.* |
| **mibot** | Did that make sense? |
| **client** | Yes. |
| **mibot** | That's great to hear, thanks for letting me know! |
| **mibot** | Now, what is the thing you like least about smoking? |
| **client** | Health issues. |
| **mibot (data)** | *You are aware of the health risks associated with smoking and are concerned about*  *your own health.* |
| **mibot** | Did what I said make sense to you? |
| **client** | Yes. |
| **mibot** | Thanks, I'm glad, this helps my listening skills |
| **mibot** | Now, what is one thing about your smoking habit that you would like to change? |
| **client** | Smoking among friends. |
| **mibot (data)** | *You would like to stop smoking among your friends.* |
| **mibot (blcr)** | *Considering you've expressed a desire to change your habit of smoking among friends, and given the prior importance you placed on health issues, it seems like you're recognizing the impact on your well-being and are looking for more health-conscious ways to socialize with friends.* |
| **mibot** | Did that make sense? |
| **client** | Yeah. |
| **mibot** | Great, let’s continue |
| **mibot** | What will it look like when you have made this change in your smoking habit? |
| **client** | Stop smoking or smoking less |
| **mibot (data)** | *You will stop smoking completely.* |
| **mibot (blcr)** | *It sounds like you are envisioning a future where you've either quit smoking entirely or reduced your smoking habits significantly, which could arguably lead to a healthier life - a concern you expressed earlier. Your desire to change the way you smoke around your friends indicates that the social aspect of your smoking habit also plays a significant role in your life, and making this change could potentially impact those interactions as well.* |
| **mibot** | Does this make sense to you? |
| **client** | Yes. |
| **mibot** | Great, this lets me know I'm on the right track |
| **mibot** | Finally, what are the steps you need to take to make this change? |
| **client** | Taking nicotine tablets. |
| **mibot (data)** | *You realize that you need to replace smoking with nicotine replacement therapy*  *(NRT).* |
| **mibot (blcr)** | *As you consider using nicotine tablets to curb your smoking, it seems that you're not only trying to lessen your health risks but also trying to change the way that smoking is intertwined with your social interactions.* |
| **mibot** | Did that make sense? |
| **client** | Yea. |
| **mibot** | Thanks, that is good to hear! |
| **mibot** | Thank you very much for this conversation, it was really helpful! |
