# Supplementary figures and images for: Generation of Backward-Looking Complex Reflections for a Motivational Interviewing–Based Smoking Cessation Chatbot Using GPT-4: Algorithm Development and Validation
Source: JMIR Ment Health. 2024 Sep 26;11:e53778. doi: 10.2196/53778 (PMC11448290; doi:10.2196/53778)

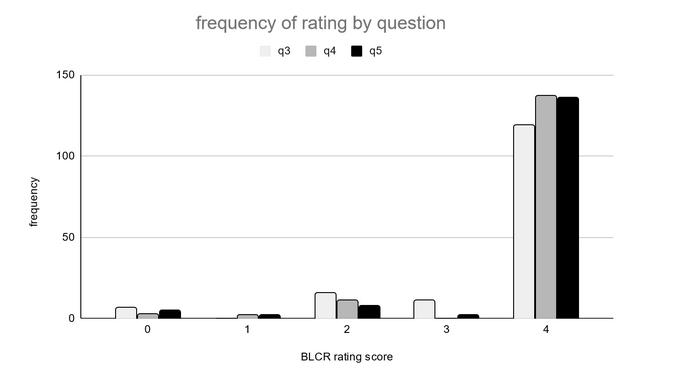

Supplement: Multimedia Appendix 2 [file mental-v11-e53778-s002.png]

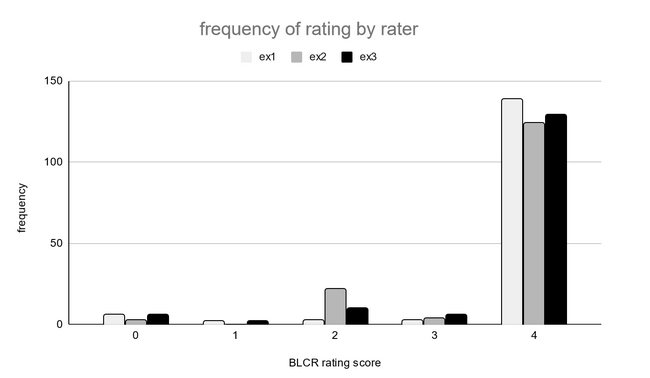

Supplement: Multimedia Appendix 3 [file mental-v11-e53778-s003.png]
